# A Survey among Plastic Surgeons Wearing a Mask in Operating Room

**DOI:** 10.29252/wjps.8.1.93

**Published:** 2019-01

**Authors:** Muhammad Ahmad, Mohammad Humayun Mohmand, Taokeer Ahmad

**Affiliations:** 1 Aesthetic Plastic Surgery & Hair Transplant Institute, Islamabad, Pakistan;; 2 Islamabad Cosmetic Surgery & Hair Transplant Institute, Islamabad, Pakistan;; 3 Peshawar, Pakistan

**Keywords:** Plastic surgeon, Wear, Mask, Operating room

## Abstract

**BACKGROUND:**

Face mask is considered to be an integral part of a surgeon’s dress in operating room. The following study was carried out among the plastic surgeons to know their views about the wearing the face masks in operating room (OR).

**METHODS:**

A questionnaire was developed and was sent to the 2 groups of plastic surgeons which included 8 questions. Group A consisted of 100 plastic surgeons from the subcontinent. Group B consisted of 100 plastic surgeons of USA and European origins. The questionnaires were sent by emails and the data was analyzed. The questionnaire was consisted of 8 questions.

**RESULTS:**

About 93% of the plastic surgeons in group A wore the mask and 86% in group B. About 96% of plastic surgeon in group A and 99% in group B used disposable masks and only 4% in group A and 1% in group B used re-usable/washable face masks. About 59% in group A and 63% in group B covered the nose. Botox and filler injections were the commonest procedures in which the surgeons opted to perform without face mask (74% in group A and 68% in group B), followed by liposuction (41% in group A and 34% in group B). The majority in both groups believed that face mask decreases the surgical site infection.

**CONCLUSION:**

Most of the plastic surgeons wore the face masks in the OR. Care must be taken to ensure that properly designed studies that determine if surgical masks prevent post-operative wound infection.

## INTRODUCTION

Face mask is considered to be an integral part of a surgeon’s dress in operating room (OR). Mikulicz in 1893 was the first surgeon to wear a mask to protect wounds from mouth bacteria.^1^ Fourteen years later, masks were regarded as optional and by 1920’s, these became accepted part of the surgeon’s uniform.^1^ These masks are thought to protect the surgical site infection by filtering oral microorganisms and prevent their spread. The benefits of wearing face mask have shown a conflicting data.^2^^-^^5^ Face shields offer more personal protection and are even more comfortable than face masks.^6^

In a survey conducted in Alberta physicians in 2007, the older physicians believed that face masks were useful in preventing the spread of the disease, whereas younger physicians were of the view that face shields should be used.^7^ Similarly, the study of 2 years by Tunevall *et al. *revealed that there was no significant difference of infection rate in 3000 general surgery patients, half being operated with face masks (4.7%) and half without facemasks (3.5%).^8^ The following study was carried out among the plastic surgeons to know their views about the wearing the face masks in operating room.

## MATERIALS AND METHODS

**Fig. 1:**
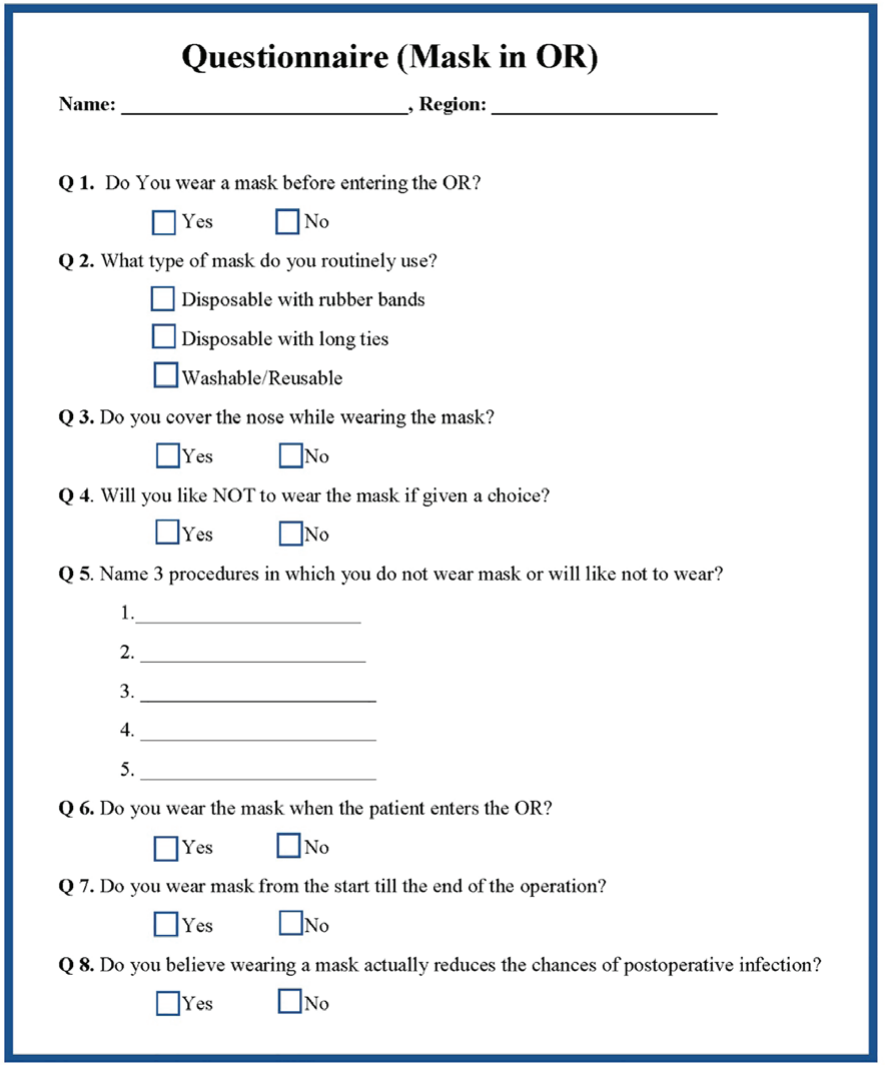
Questionnaire used in the study

A questionnaire was developed and was sent to the 2 groups of plastic surgeons (Figure 1). Group A consisted of 100 plastic surgeons from the subcontinent (i.e., Pakistan, India, Bangladesh). These were the members of local plastic surgery societies, i.e., Pakistan Association of Plastic Surgeons, Indian Association of Plastic Surgeons and Bangladesh Association of Plastic Surgeons. Group B consisted of 100 plastic surgeons of USA and European origins. These were members of American Society of Plastic Surgeons, European Association of Plastic Surgeons and American Academy of Facial Plastic Reconstructive Surgeons. The questionnaires were sent by emails. The collected data was analyzed by using the software (‘Stats Tester’ © version 2.0.1, 2016-17, BMP Group, Saitama, Japan).

## RESULTS

**Fig. 2:**
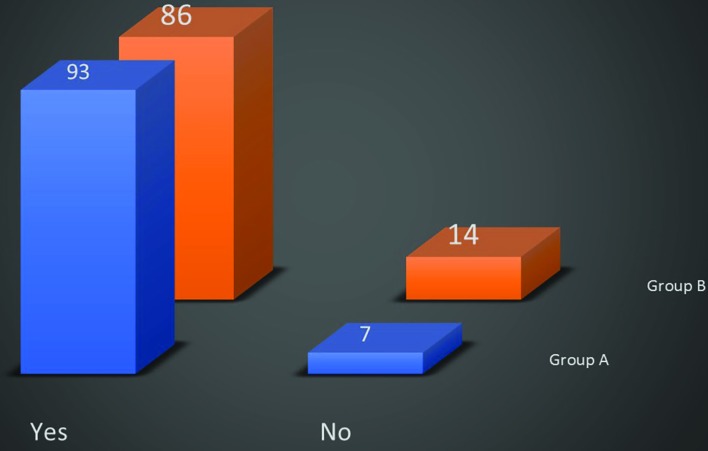
Wearing the masks

The questionnaire consisted of 8 questions. (i) Do You wear a mask before entering the OR? About 93% of the plastic surgeons in group A wore the mask and 86% in group B (Figure 2). (ii) What type of mask do you routinely use? About 96% of plastic surgeon on group A and 99% in group B used disposable masks and only 4% in group A and 1% in group B used re-usable/washable face masks. Disposable masks with rubber was used among 45 surgeons in group A and 39 in group B. These figures for disposable masks with long ties were 51 and 60 and for washable/reusable were 4 and 1, respectively. 

(iii) Do you cover the nose while wearing the mask? Totally, 59% in group A (n=59) covered their nose while operating whereas 63% in group B (n=63) covered the nose. Forty-one in group A and 37 surgeons in group B did not cover the nose. (iv) Will you like NOT to wear the mask if given a choice? About 78% in group A (n=78) and 65% in group B (n=65) voted not to wear in operating room if given a choice. These figures for YES response were 78 and 65, respectively. (v) Name 3 procedures in which you do not wear mask or will like not to wear? Botox and filler injections were the commonest procedures in which the surgeons opted to perform without face mask (74% in group A and 68% in group B), followed by liposuction (41% in group A and 34% in group B) (Table 1). 

**Table 1:** Procedures used in OR

	**Group A**	**Group B**
Botox/Filler injection	74	68
Liposuction	41	34
Hair transplant	36	30
Nose reshaping	20	11
Face lift	8	4
Local anaesthesia case	9	4
Always wear	19	11

(vi) Do you wear the mask when the patient enters the OR? Only 31% in group A and 24% in group B used to wear mask when the patients enter the operating room (Figure 3). (vii) Do you wear mask from the start till the end of the operation? Only 14% in group A and 7% in group B used to wear the mask from start till the patient goes out of the OR (Figure 4). (viii) Do you believe wearing a mask actually reduces the chances of postoperative infection? About 91% in group A and 82% in group B believed that face mask results in decreased chances of patient infection or surgical site infection (Figure 5).

**Fig. 3:**
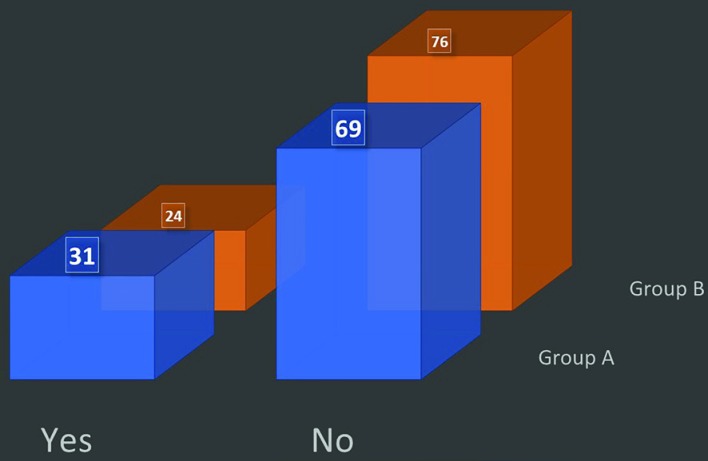
Time of mask wearing

**Fig. 4:**
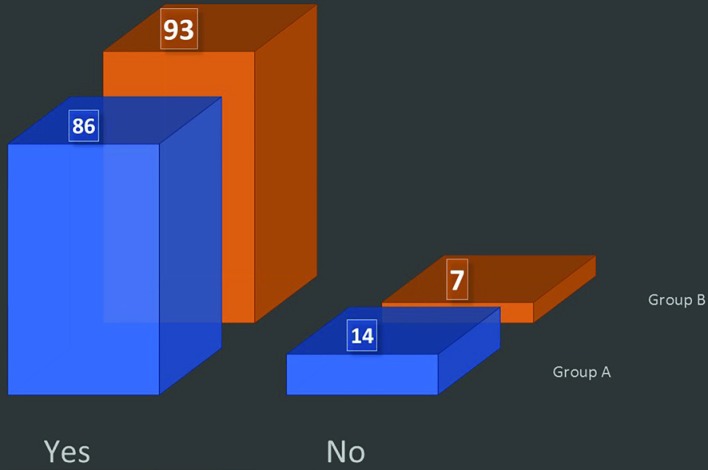
Duration of mask wearing

**Fig. 5:**
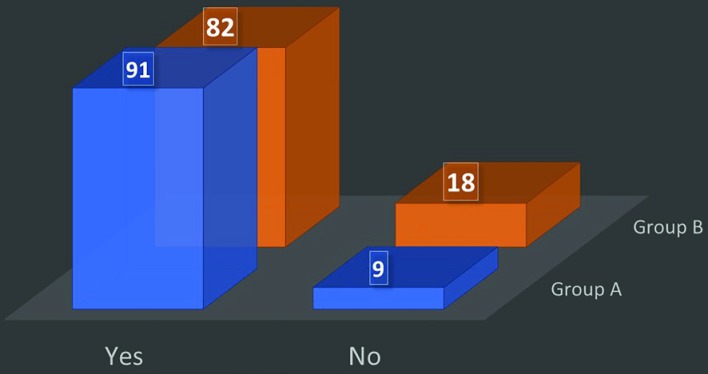
Belief about mask

## DISCUSSION

Masks were introduced approximately a century ago to protect patients from surgical site infection.^1^ Masks were questioned in a study by Orr in 1981 which illustrated that operations carried out in OR in which hospital personnel wore no masks showed no increase in post-surgical infection rates to the patients from OR in which masks were worn.^9^ The study by Tunevall *et al.* analyzed the link between wearing surgical masks and post-surgical infection.^8^ The study found the infection rate was 4.7% with masks and 3.5% without the masks and there was no increase in surgical site infections when masks were not worn.^8^

A similar observation was noted by Ruthman *et al.* in which no difference was observed in infection rates based on wound sutured in emergency room regardless of masks being worn or not.^10^ An interesting observation was noted by Lipp *et al.* that masks can actually contribute to wound infection, though venting or leaking of air through the side of the masks or by being worn or removed incorrectly.^11^ Wearing one masks all day long or wearing a wet mask is useless for preventing cross contamination.^12^ Moreover, surgical masks protect hospital staff from possible infection from the patients. According to operating room nurses of Association of Canada that ‘all persons entering the surgical site should wear a mask when open sterile items and equipment are present.^13^

Davis *et al.* suggested that ethically it is the responsibility of the physician to obtain informed consent to perform a sterile procedure or surgery without a mask.^7^ The present study basically gave the general view of plastic surgeons about the practice of use of the masks in operating room. We analyzed a few interesting points, for example, more than one third of the surgeons were of the practice of not covering the nose while wearing the mask in OR and about two thirds wished not to wear the mask if given the choice. The reasons could be personal to many surgeons but generally surgeons wearing spectacles find it difficult to couple with the fog effect while breathing in the mask especially if the nose is also covered. Sometimes the glasses of the spectacles were hidden by the masks in the lower part obstructing the field of vision.

Majority of the surgeons in each group did not wear the mask when patient enters the OR which alleviates the anxiety of the patients, by adding the recognizable faces in the hostile environment of OR. The study by Webster *et al.* indicated that surgical site infection rates did not increase when non-scrubbed OR personnel did not wear a face mask.^14^ Surprisingly, the majority of the plastic surgeons in the present study were of the belief that masks actually reduce the surgical site infection. The evidence to support the continued use of masks is limited yet most guidelines for dress codes continue to recommend their use. More research must be undertaken before a definitive conclusion can be made. Care must be taken to ensure that properly designed studies that determine if surgical masks prevent post-operative wound infection. 

## CONFLICT OF INTEREST

The authors declare no conflict of interest.
